# Luminal progenitor and mature cells are more susceptible than basal cells to radiation-induced DNA double-strand breaks in rat mammary tissue

**DOI:** 10.1093/jrr/rrae067

**Published:** 2024-09-05

**Authors:** Kento Nagata, Mayumi Nishimura, Kazuhiro Daino, Yukiko Nishimura, Yuya Hattori, Ritsuko Watanabe, Daisuke Iizuka, Akinari Yokoya, Keiji Suzuki, Shizuko Kakinuma, Tatsuhiko Imaoka

**Affiliations:** Department of Radiation Effects Research, Institute for Radiological Science, National Institutes for Quantum Science and Technology, 4–9–1 Anagawa, Inage-ku, Chiba 263-8555, Japan; Department of Radiation Effects Research, Institute for Radiological Science, National Institutes for Quantum Science and Technology, 4–9–1 Anagawa, Inage-ku, Chiba 263-8555, Japan; Department of Radiation Effects Research, Institute for Radiological Science, National Institutes for Quantum Science and Technology, 4–9–1 Anagawa, Inage-ku, Chiba 263-8555, Japan; Department of Radiation Effects Research, Institute for Radiological Science, National Institutes for Quantum Science and Technology, 4–9–1 Anagawa, Inage-ku, Chiba 263-8555, Japan; Department of Electrical Engineering and Information Science, Faculty of Electrical Engineering and Information Science, National Institute of Technology Kure College, 2–2–11 Aga-minami, Kure, Hiroshima 737-8506, Japan; Institute for Quantum Life Science, National Institutes for Quantum Science and Technology, 4–9–1 Anagawa, Inage-ku, Chiba 263-8555, Japan; Department of Radiation Effects Research, Institute for Radiological Science, National Institutes for Quantum Science and Technology, 4–9–1 Anagawa, Inage-ku, Chiba 263-8555, Japan; Institute for Quantum Life Science, National Institutes for Quantum Science and Technology, 4–9–1 Anagawa, Inage-ku, Chiba 263-8555, Japan; Department of Radiation Medical Sciences, Atomic Bomb Disease Institute, Nagasaki University, 1–12–4 Sakamoto, Nagasaki 852-8523, Japan; Department of Radiation Effects Research, Institute for Radiological Science, National Institutes for Quantum Science and Technology, 4–9–1 Anagawa, Inage-ku, Chiba 263-8555, Japan; Department of Radiation Effects Research, Institute for Radiological Science, National Institutes for Quantum Science and Technology, 4–9–1 Anagawa, Inage-ku, Chiba 263-8555, Japan

**Keywords:** mammary gland, DNA double-strand break, stem/progenitor cell, radiation

## Abstract

Ionizing radiation promotes mammary carcinogenesis. Induction of DNA double-strand breaks (DSBs) is the initial event after radiation exposure, which can potentially lead to carcinogenesis, but the dynamics of DSB induction and repair are not well understood at the tissue level. In this study, we used female rats, which have been recognized as a useful experimental model for studying radiation effects on the mammary gland. We focused on differences in DSB kinetics among basal cells, luminal progenitor and mature cells in different parts of the mammary duct. 53BP1 foci were used as surrogate markers of DSBs, and 53BP1 foci in each mammary epithelial cell in immunostained tissue sections were counted 1–24 h after irradiation and fitted to an exponential function of time. Basal cells were identified as cytokeratin (CK) 14^+^ cells, luminal progenitor cells as CK8 + 18^low^ cells and luminal mature cells as CK8 + 18^high^ cells. The number of DSBs per nucleus tended to be higher in luminal cells than basal cells at 1 h post-irradiation. A model analysis indicated that basal cells in terminal end buds (TEBs), which constitute the leading edge of the mammary duct, had significantly fewer initial DSBs than the two types of luminal cells, and there was no significant difference in initial amount among the cell types in the subtending duct. The repair rate did not differ among mammary epithelial cell types or their locations. Thus, luminal progenitor and mature cells are more susceptible to radiation-induced DSBs than are basal cells in TEBs.

## INTRODUCTION

Ionizing radiation increases human morbidity and mortality due to carcinogenesis, as revealed by epidemiological studies, including those of Japanese atomic-bomb survivors [1]. The effects of ionizing radiation on living organisms are mainly initiated by damage to DNA, which can affect a single strand or both strands of double-stranded DNA [2]. DNA double-strand breaks (DSBs) constitute one of the most serious forms of DNA damage, activating various cellular signaling pathways. The tumor-suppressor protein p53-binding protein 1 (53BP1) initiates nonhomologous end-joining repair of DSBs [3]. 53BP1 accumulates at almost all DSB sites, and therefore its localization as small spherical signals in the cell nucleus (often called foci) is widely used as a marker of DSBs [3]. As previous studies have indicated that induction of DSBs should be the key event related to radiation-induced carcinogenesis, it is essential to investigate the kinetics of DSBs in cells of origin of cancer. In general, well-differentiated, nonproliferating cells are resistant to radiation, whereas actively proliferating cells such as subsets of somatic stem and progenitor cells are more sensitive [4]. Previous studies have reported that the cellular differentiation state also affects the repair rate of DSBs [5, 6]. If DNA damage in stem and progenitor cells is erroneously repaired and mutations accumulate, these cells can serve as the cells of origin for cancer [7]. Therefore, elucidation of cellular DNA repair dynamics in the early period after radiation exposure may contribute to understanding the mechanism of carcinogenesis.

The risk of radiation-related cancer is higher in the mammary gland than in most other organs [8–11]. The risk of radiation-related breast cancer is particularly high after radiation exposure during puberty [12, 13]. Some studies have used rats as an animal model for human breast cancer because of their hormone-dependent nature of carcinogenesis and similar pathological characteristics [14, 15]. The mammary epithelium consists mainly of two layers of cells, namely basal cells (mostly myoepithelial cells) and luminal cells [16]. Cell-lineage tracing and immunostaining studies in mice have shown that basal cells express cytokeratin (CK) 14 and luminal progenitor cells express both CK8 and CK18, whereas the luminal mature cells express CK18 but not CK8 [17]. Thus, these CKs can serve as markers of those cell types. CK comprises a family of intermediate filaments that form the cytoskeleton of epithelial cells. Post-pubertal mammary ducts increase in mass via cell proliferation in the terminal end bud (TEB), which is a club-like structure at the end of each mammary duct and extend into the subcutaneous fat tissue [18]. The TEB is **s**urrounded by a single layer of basal cells, and multilayered luminal cells are placed inside; in contrast, mammary ducts are composed of single layers of outer basal and inner luminal cells [18]. TEBs are of particular interest in terms of their unique properties including high rates of proliferation (60–90%) and apoptosis (5–15%), invasive capacity, angiogenic properties and ability to recruit stromal cells [18]. As such, it has long been known that cells present in TEBs can serve as the origin of carcinogenesis [19].

The amount of radiation-induced DSBs and their repair rates have been analyzed in female pre- and postmenopausal mammary tissue [20]. A recent flow cytometry study of rats showed that the initial amount of radiation-induced DSBs differs among mammary epithelial cell types [21]. Results of an experimental system using cultured human mammary cells suggested that the response to oxidative stress after radiation exposure differs among epithelial cell types [20]. Nevertheless, whether the initial DSB burden and its repair rate vary by cell type (i.e. basal vs luminal, progenitor vs mature) and location in the gland (i.e. TEB vs other parts of the mammary duct) is not completely understood. However, evidence suggests that mammary epithelial cell types differ with respect to metabolic activities [22–24], and thus it is possible that the response to radiation exposure may differ among these cell types and their locations in the mammary gland. To investigate this possibility, we used a rat model of mammary carcinogenesis to evaluate the induction and repair of DSBs after radiation exposure as stratified by cell type and location.

## MATERIALS AND METHODS

### Animals

Female virgin Sprague-Dawley rats (Jcl:SD; CLEA Japan Inc., Tokyo, Japan), purchased at 5 weeks of age, were either left unirradiated or subjected to whole body irradiation with 1 Gy of ^137^Cs γ-rays (dose-rate, 0.4 Gy/min) using a Gammacell 40 irradiator (Nordion, Ottawa, Canada) between 09:00 and 12:00 at 7 weeks of age. Vaginal smears were taken daily over at least one estrous cycle to confirm their cycling. All experiments used postpubertal rats in estrus. All rats were fed a CE-2 diet (CLEA Japan) and provided chlorinated, acidified water ad libitum. They were maintained under a specific pathogen-free condition in autoclaved cages on a 12-h light/12-h dark cycle. All animal experiments were approved by the Institutional Animal Care and Use Committee of the National Institutes for Quantum Science and Technology (Approval No. 19-1003) and were performed in accordance with the Fundamental Guidelines for Proper Conduct of Animal Experiment and Related Activities in Academic Research Institutions under the jurisdiction of the Ministry of Education, Culture, Sports, Science and Technology of Japan.

### Preparation of histological sections

Rats were euthanized by exsanguination under isoflurane-mediated deep anesthesia (4% in air). The fourth mammary gland of 7-week-old rats was then collected, extended on glass slides and fixed in 10% phosphate-buffered formalin. Paraffin-embedded sections (3 μm) were prepared with a microtome (Leica Microsystems GmbH, Wetzlar, Germany) and deparaffinized in xylene, rehydrated in graded ethanol and then subjected to antigen retrieval by microwaving at 95°C in 10 mM Tris–HCl buffer (pH 9.0) for 40 min for multiple immunostains or autoclaving at 121°C in 10 mM citrate buffer (pH 6.0) for stains of 8-hydroxydeoxyguanosine (8-OHdG). For multiple immunostains with CK marker, DSB marker and proliferation marker, these sections were further treated with a cocktail of primary antibodies in a blocking solution (Protein Block, Agilent Technology, Santa Clara, CA) at 37°C for 2 h (Table 1). Thereafter, the sections were rinsed three times in Tris-buffered saline (50 mM Tris–HCl, pH 7.6) with 0.05% (w/v) Tween 20 (TBST) and then treated with a cocktail of secondary antibodies at room temperature for 1 h (Table 1). The sections were then rinsed three times in the same buffer and mounted with Vectashield mounting medium containing 4′,6-diamidino-2-phenylindole (DAPI) (Vector Laboratories, Burlingame, CA). For staining of 8-OHdG, sections were further treated with a primary antibody in a blocking solution at 4°C overnight (Table 1). The sections were rinsed three times in TBST and treated with secondary antibody (Histofine Simple Stain AP (M), Nichirei Biosciences Inc., Tokyo, Japan) at room temperature for 30 min. Thereafter, the sections were rinsed three times in TBST and treated with chromogenic reagent (Histofine First Red II substrate kit, Nichirei Biosciences). The specimens were then counter stained with hematoxylin, air-dried, cleared in xylene and mounted.

**Table 1:** Antibody list

Antigen	Clone	Label	Supplier	Catalog number	Species	Dilution
*Primary antibody*						
CK14	LL002	None	Abcam	ab7800	Mouse	1:500
CK8 + 18	Polyclonal	None	Progen	GP11	Guinea pig	1:500
CK18	Polyclonal	None	Abcam	ab52948	Rabbit	1:500
Ki-67	SP6	None	Invitrogen	MA5-14520	Rabbit	1:100
53BP1	Polyclonal	None	Bethyl	A300-272A	Rabbit	1:500
8 OHdG	N45.1	None	JalCA	MOG-020P	Mouse	1:50
*Secondary antibody*						
Rabbit IgG	Polyclonal	AF594	Abcam	ab150088	Goat	1:500
Mouse IgG	Polyclonal	AF488	Abcam	ab150117	Goat	1:500
Guinea pig IgG	Polyclonal	AF647	Abcam	ab150187	Goat	1:500

AF = Alexa Fluor, IgG = immunoglobulin G.

### Cell counting

Fluorescence images were obtained using a Disk Scanning Unit Confocal Microscopy system (Olympus, Tokyo, Japan) mounted on an IX83 inverted microscope (Olympus). ImageJ (National Institutes of Health, Bethesda, MD) was used to concatenate and segment the images. Statistical analysis of numerical values was performed using R (version 4. 1. 1) (https://www.R-project.org/). For analysis of cell numbers, cells were counted in TEBs and their subtending duct per animal. Differences between data from different post-irradiation time points were assessed by analysis of variance (ANOVA) followed by Dunnett’s tests. Comparison of cell number between TEB and its subtending duct was performed by three-way ANOVA (on cell type, location and individual) followed by paired *t*-tests. For analysis of foci numbers, foci in all cell nuclei in the analysis above were counted, and the average value was considered representative of an individual rat. The differences in the number of foci per cell type and location in the gland were analyzed using three-way ANOVA (on cell type, location and time after exposure) or a two-way ANOVA (on time after exposure and individual). The Dunnett test was used for multiple comparisons after ANOVA.

The number of animals used was three to six in total as described in Figs 2 and 4. The experiments were performed in triplicate (animals, *n* = 1–2 each, were purchased at three separate times); for 1 h post-irradiation, the irradiation was performed two times for different sets of animals, which were purchased at one time, with their batches mixed by the breeder beforehand.

### Regression analysis

To evaluate the DSB repair kinetics in different cell types (basal cells, luminal progenitor cells and luminal mature cells) and locations (TEBs and subtending ducts), we used 53BP1 foci number data for each cell type and location for a least-squares fitting to the following exponential function using R. First, the following formula was used to obtain an exponential equation for a specified cell type in a specified location:


(1) \begin{equation*}F(t)=A{e}^{- Bt} \end{equation*}

where $t$ is the time (h) after exposure, $F(t)$ is the number of foci per nuclear section at time $t$, $A$ represents the initial number of foci per nuclear section and $B$ represents the decay factor of the reciprocal of time (h^−1^), which was presented as a coefficient indicating the speed of DSB repair within 24 h after radiation exposure. To assess the statistical significance of differences in parameters $A$ and $B$ between the cell types or between locations, these parameters were expressed as follows:


(2) \begin{equation*} \left\{\begin{array}{@{}c}A={A}_{\mathrm{BC}}+{a}_{\mathrm{LPC}-\mathrm{BC}}\cdot {I}_{\mathrm{LPC}}+{a}_{\mathrm{LMC}-\mathrm{BC}}\cdot {I}_{\mathrm{LMC}}\\ {}B={B}_{\mathrm{BC}}+{b}_{\mathrm{LPC}-\mathrm{BC}}\cdot {I}_{\mathrm{LPC}}+{b}_{\mathrm{LMC}-\mathrm{BC}}\cdot {I}_{\mathrm{LMC}}\end{array}\right. \end{equation*}

(3) \begin{equation*} \left\{\begin{array}{@{}c}A={A}_{\mathrm{LPC}}+{a}_{\mathrm{LMC}-\mathrm{LPC}}\cdot {I}_{\mathrm{LMC}}+{a}_{\mathrm{BC}-\mathrm{LPC}}\cdot {I}_{\mathrm{BC}}\\ {}B={B}_{\mathrm{LPC}}+{b}_{\mathrm{LMC}-\mathrm{LPC}}\cdot {I}_{\mathrm{LMC}}+{b}_{\mathrm{BC}-\mathrm{LPC}}\cdot {I}_{\mathrm{BC}}\end{array}\right. \end{equation*}

(4) \begin{equation*} \left\{\begin{array}{@{}c}A={A}_{\mathrm{TEB}}+{a}_{\mathrm{SD}-\mathrm{TEB}}\cdot {I}_{\mathrm{SD}}\\ {}B={B}_{\mathrm{TEB}}+{b}_{\mathrm{SD}-\mathrm{TEB}}\cdot {I}_{\mathrm{SD}}\end{array}\right. \end{equation*}

where subscripts BC, LPC, LMC, TEB and SD indicate basal, luminal progenitor, luminal mature cells, TEBs and subtending ducts, respectively, ${A}_X$ and ${B}_X$ indicate the initial value and the decay factor, respectively, for cell type or location $X$, ${I}_X$ is a dummy variable for cell type or location $X$ and ${a}_{X-Y}$ and ${b}_{X-Y}$ are the difference in $A$ and $B$, respectively, between cell types or locations $X$ and $Y$. A difference between $X$ and $Y$ was considered statistically significant if ${a}_{X-Y}$ or ${b}_{X-Y}$ was significantly larger or smaller than zero.

## RESULTS

### Cytokeratin expression correlates with certain features of basal cells, luminal progenitor cells and luminal mature cells of the rat mammary epithelium

**Fig. 1:**
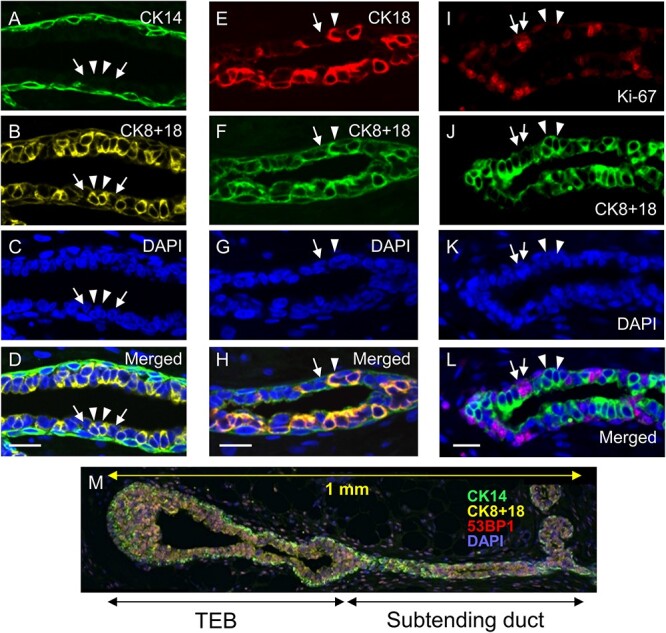
Immunostaining for CKs and the proliferating cell marker Ki67 in rat mammary tissues. All colors are pseudocolors. CK14 and CK8 + 18. (**A**) Basal cells expressing CK14 demarcate the mammary duct. (**B**) Luminal cells in the inner layer are recognized by anti-CK8 + 18. (**C**) DAPI-stained cell nuclei. (**D**) Overlay of A–C. Arrowheads, CK8 + 18^high^. Arrows, CK8 + 18^low^. Scale bar, 25 μm. CK18 and CK8 + 18. (**E**) Luminal mature cells expressing CK18 (arrowhead). (**F**) Luminal cells were recognized by anti-CK8 + 18. (**G**) DAPI-stained cell nuclei. (**H**) Overlay of E–G. Arrowhead, CK18^+^/CK8 + 18^high^. Arrow, CK18^−^/CK8 + 18^low^. Scale bar, 25 μm. Ki67 and CK8 + 18. (**I**) Proliferation marker Ki67. (**J**) Luminal cells were recognized by anti-CK8 + 18. (**K**) DAPI-stained cell nuclei. (**L**) Overlay of I–K. Arrowheads, Ki67^−^/CK8 + 18^low^. Arrows, Ki67^+^/CK8 + 18^low^. Scale bar, 25 μm. (**M**) A structure of TEB and its subtending duct. From the terminus of a mammary duct, the first 0.5 mm was defined as the TEB and the next 0.5 mm as a subtending duct.

To identify basal cells, luminal progenitor cells and luminal mature cells of the rat mammary epithelium on paraffin tissue sections, the expression of CKs and the cell-proliferation marker Ki-67 was quantified by immunofluorescence staining. The mammary epithelium comprises one layer of basal cells and one or more layers of luminal cells [21]. CK14 was expressed in basal cells, whereas CK8 and CK18 (designated CK8 + 18) were expressed in luminal cells (Fig. 1A–D), as reported previously in mice and rats [17, 21]. Some luminal cells were strongly positive for CK8 + 18 (Fig. 1B, arrowheads; CK8 + 18^high^), whereas others were weakly positive (Fig. 1B, arrows; CK8 + 18^low^). This difference in staining was further examined by co-staining with an antibody that recognizes only CK18, revealing that fluorescence from CK18^+^ cells coincided with that of CK8 + 18^high^ cells and that fluorescence from CK18^−^ cells coincided with that of CK8 + 18^low^ cells (Fig. 1E–H). Of note, Ki-67 was expressed only in a subset of CK8 + 18^low^ cells and not at all in CK8 + 18^high^ cells (Fig. 1I–L). Thus, we defined CK14^+^, CK8 + 18^low^ and CK8 + 18^high^ cells as basal, luminal progenitor and luminal mature cells, respectively.

### Cell number remains stable after radiation exposure except for a transient increase in luminal progenitor cells

**Fig. 2:**
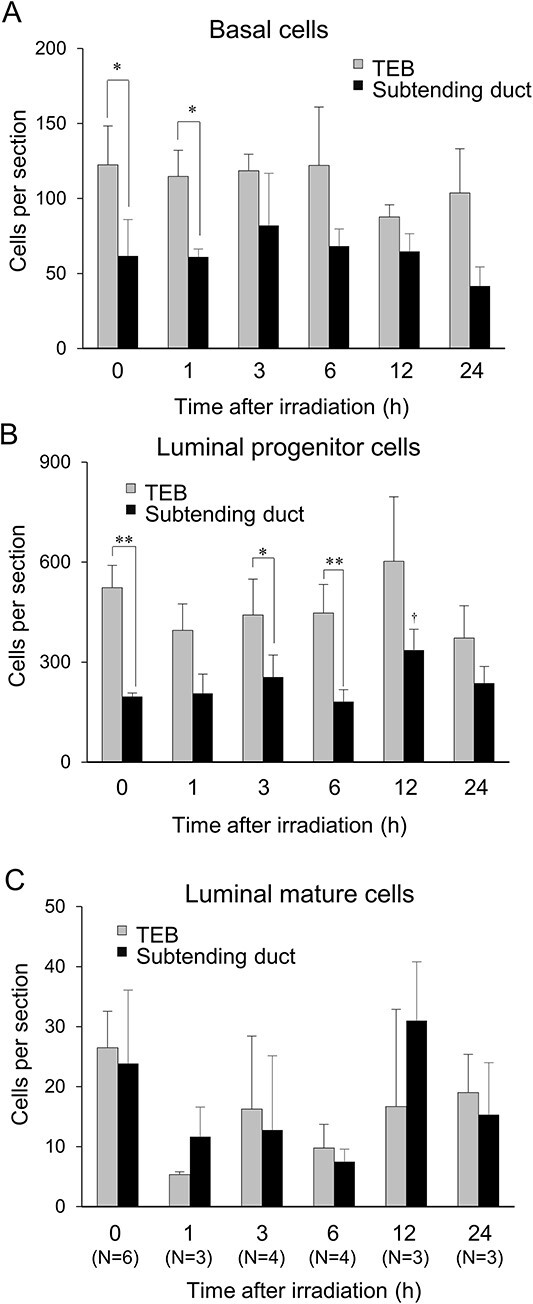
Quantification of cells in the mammary gland. Time series of the number of basal cells (**A**), luminal progenitor cells (**B**) and luminal mature cells (**C**). Vertical bars, SD (*n* = 3–6 rats). ^*^P<0.05, ^*^^*^P<0.01 between locations by ANOVA followed by the paired *t*-test. ^†^*P*<0.05 vs 0 Gy by Dunnett’s test.

**Fig. 3:**
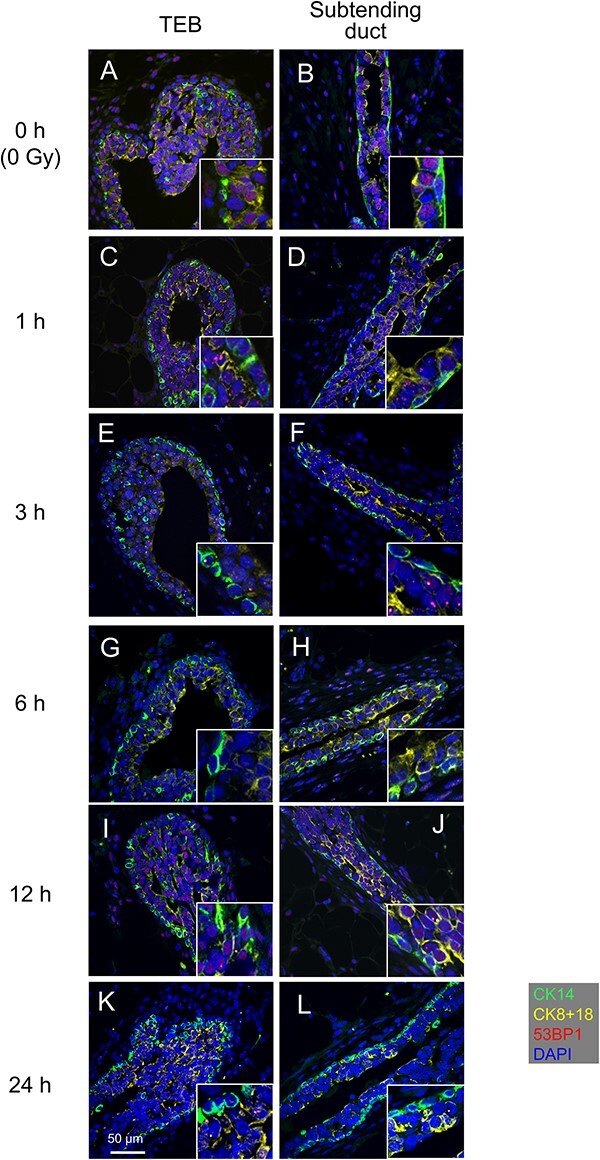
Immunohistochemical analysis of the rat mammary gland before and after γ-irradiation. Immunostaining with antibodies specific for CK14, CK8 + 18 and 53BP1 of a TEB (**A**, **C**, **E**, **G**, **I** and **K**) and a subtending duct (**B**, **D**, **F**, **H**, **J** and **L**) of the mammary gland of nonirradiated rats (A, B) and irradiated rats at 1 h (C, D), 3 h (E, F), 6 h (G, H), 12 h (I, J) and 24 h (K, L) after irradiation. Scale bar, 50 μm.

To quantify the three mammary epithelial cell types at each location in the gland, we divided the first 1.0 mm of the mammary ductal terminus into two regions, namely the TEB (0–0.5 mm) and the subtending duct (0.5–1.0 mm) (Fig. 1M). The abundance of each of these three cell types was determined in each region. In the mammary gland of nonirradiated rats, luminal progenitor cells were most abundant, followed by basal cells, in both the TEB and subtending duct (Figs 2 and 3). In the gland of γ-irradiated (1 Gy) rats, the numbers of the three cell types in each of the TEB and the subtending duct did not differ up to 24 h post-irradiation, except that the luminal progenitor cells increased 12 h after exposure (Fig. 2B). Thus, the luminal progenitor cells were major in TEBs (0–0.5 mm) and subtending ducts (0.5–1.0 mm) of the mammary epithelium.

### Induction of 53BP1 foci by radiation exposure and their steady decrease in all mammary epithelial cell types of the terminal end bud and subtending duct

**Fig. 4:**
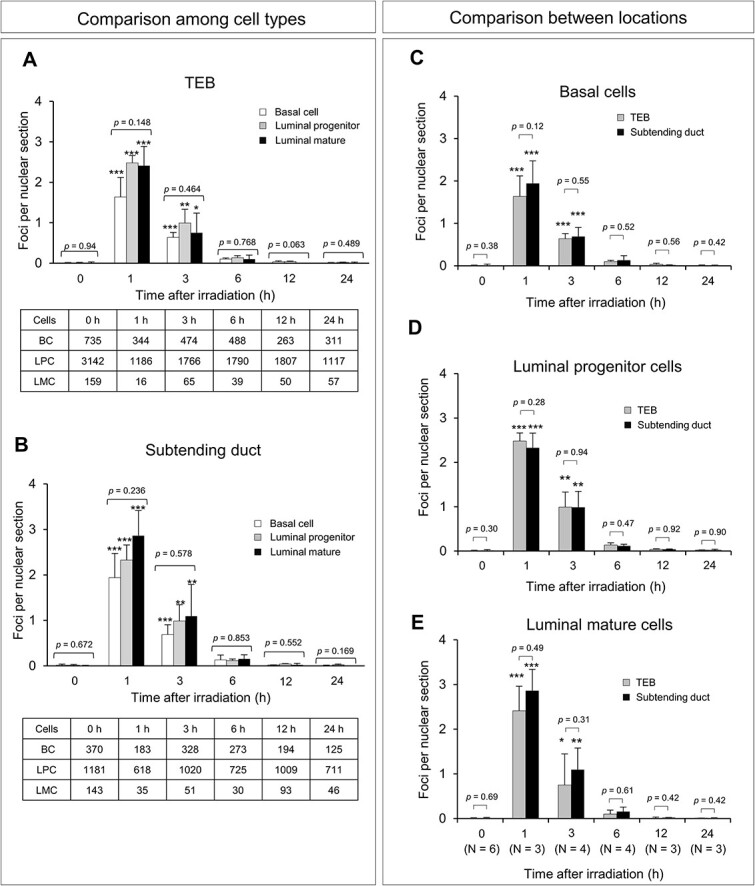
Temporal change in 53BP1 foci abundance before and after γ-irradiation. Number of 53BP1 foci in the TEB (**A**) and subtending duct (**B**) by cell type. The tables show the total number of cells counted. The data in A and B were rearranged to indicate the number of foci in basal cells (**C**), luminal progenitor cells (**D**) and luminal mature cells (**E**) by location in the gland. Vertical bars, SD (*n* = 3–6 rats). ^*^P<0.05, ^*^^*^P<0.01, ^*^^*^^*^P<0.001 vs 0 Gy by Dunnett’s test. The *P* values directly shown in the graphs were calculated by one-way ANOVA (A and B) or Student’s *t*-test (C–E).

Next, to analyze the induction and repair of DSBs, immunofluorescence staining was carried out for 53BP1 (marker of DSBs) and for CK14 and CK8 + 18, which discriminate between basal, luminal progenitor and luminal mature cells (Fig. 3). First, to determine the rate of DSB repair prior to radiation exposure, the number of 53BP1 foci per cell nucleus was counted in confocal images of mammary tissues from nonirradiated rats, indicating no difference in DSB repair rate among cell types or between locations in the gland (BC in TEB, 0.0062 ± 0.0099; BC in subtending duct, 0.013 ± 0.026; LPC in TEB, 0.0088 ± 0.0107; LPC in subtending duct, 0.014 ± 0.019; LMC in TEB, 0.0093 ± 0.0207; LMC in subtending duct, 0.005 ± 0.010; mean ± SD) (Figs 3A and B and 4). We then assessed temporal changes in DSB repair after irradiation. At 1 h post-irradiation, 53BP1 foci appeared in the nucleus as dot-like structures in each of the three cell types (Fig. 3C and D). The 53BP1 foci were also observed at 3, 6 and 12 h post-irradiation, with their numbers clearly decreasing with time (Fig. 3E–J). By 24 h post-irradiation, the foci had almost disappeared (Fig. 3K and L).

We then compared the number of 53BP1 foci per nuclear section among the three cell types. At 1 h post-irradiation, significantly more 53BP1 foci were induced in basal, luminal progenitor and luminal mature cells of both the TEB and subtending duct compared with nonirradiated rats, and the number of foci continued to increase until 3 h post-exposure depending on the cell type and location (Fig. 4A and B). By 6, 12 and 24 h after irradiation, the number of foci did not differ significantly in any of the cell types at each of the two locations, approaching the level observed for nonirradiated cells (Fig. 4A and B). Moreover, the number of foci in the three cell types did not differ significantly in either the TEB or subtending duct, except between luminal progenitor (0.042 foci) and mature (0 foci) cells at 12 h in the TEB (Fig. 4A); however, this difference was due to the very small number of cells counted in luminal mature cells (Fig. 2C) and hence may be biologically nonmeaningful. Although not statistically significant, the number of 53BP1 foci in basal cells tended to be lower than that of luminal cells (progenitor and mature) at 1 h post-irradiation, regardless of the location in the gland (Fig. 4A and B). No significant difference was observed between the locations in the gland for any cell type at any time point (Fig. 4C–E). Overall, these results indicated that the number of radiation-induced DSBs decreased steadily between 1 and 24 h post-exposure with similar kinetics in both the TEB and subtending duct, with possibly fewer initial DSBs in basal cells.

### Fitting to exponential functions supports cellular heterogeneity in the initial numbers of double-strand breaks and concordance with the repair rate

**Fig. 5:**
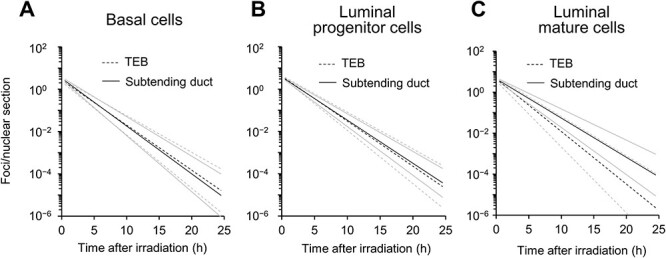
Predicted decay in the number of foci. The parameter estimates in Table 2 were substituted into Equation (1), and the obtained values were plotted on the vertical axis (which is logarithmic) with the time after radiation exposure on the horizontal axis. (**A**) Basal cells. (**B**) Luminal progenitor cells. (**C**) Luminal mature cells. Black lines use point estimates, whereas grey lines use point estimates ± fitting errors.

To analyze the kinetics of radiation-induced DSB formation and assess possible differences among the three cell types, an exponential equation (Equation (1)) was fitted to the 53BP1 foci data (Fig. 4). The parameter estimates support the aforementioned tendency of slight initial post-irradiation damage in the TEB, with similar decay factors (i.e. repair rates) in all cell types and locations (Table 2). Data obtained with Equations (2) and (3) revealed that basal cells had significantly less initial damage than luminal progenitor and mature cells in the TEB (*P* = 0.02), whereas no significant difference in decay factors was apparent among cell types (Table 2, Fig. 5). In contrast, the initial damage and the decay factor did not differ significantly among cell types in the subtending duct. Data obtained with Equation (4) revealed no significant difference in initial damage or decay factor between the TEB and subtending duct for any cell type. Thus, this analysis indicated that fewer DSB occurred in basal cells than in the two luminal cell types in the TEB but not subtending duct.

**Table 2:** Regression analysis of DSB repair kinetics (mean ± SE)

Cell type	Location	Initial damage^a^ (foci per nuclear section) (${A}$)	Decay factor (h^−1^) (${B}$)
Basal cell	TEB	2.68 ± 0.41^b^^,^^c^	0.49 ± 0.09
	Subtending duct	3.27 ± 0.43	0.52 ± 0.09
Luminal progenitor cell	TEB	4.05 ± 0.37^b^	0.49 ± 0.06
	Subtending duct	3.74 ± 0.35	0.47 ± 0.06
Luminal mature cell	TEB	4.37 ± 0.88^c^	0.59 ± 0.15
	Subtending duct	4.42 ± 0.62	0.44 ± 0.09

^a^Extrapolation to 0 h after 1-Gy ^137^Cs γ irradiation

^b^ *P*=0.023 between basal and luminal progenitor cells in TEB

^c^ *P*=0.018 between basal and luminal mature cells in TEB

### Basal cells maintain lower oxidative stress than luminal cells

**Fig. 6:**
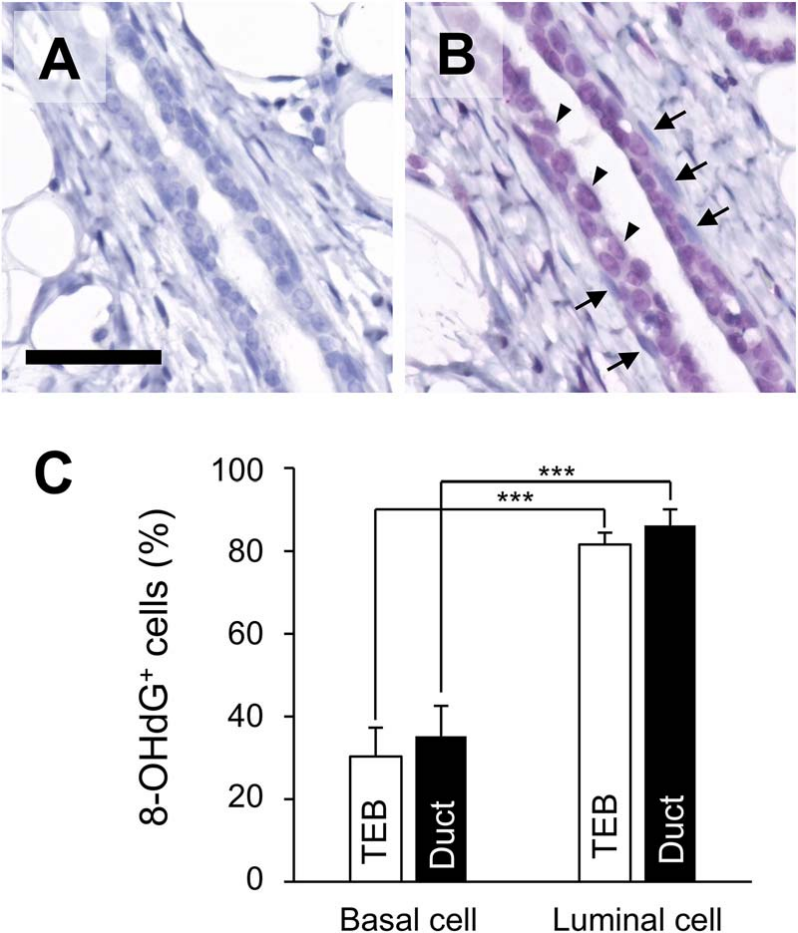
Immunostaining results of 8-OHdG in non-irradiated tissue. (**A**) Negative control without the primary antibody. (**B**) Detection of 8-OHdG by antibody reaction. Shown are representative histological images of the three samples used in the analysis. The arrows indicate basal cells without 8-OHdG, while the arrowheads indicate luminal cells with 8-OHdG (Fast Red staining with hematoxylin counterstaining). Scale bar, 50 μm. (**C**) Quantitative analysis of 8-OHdG-positive cells in basal and luminal cells. Vertical bars, SD (*n* = 3 rats). ^*^^*^^*^P<0.001 by Student’s *t*-test.

We hypothesized that the basal cells were in an antioxidant state, which explains why the number of DSBs revealed by immunostaining and subsequent fitting analysis differed between basal and luminal cells. Therefore, we evaluated DNA oxidative stress in basal and luminal cells. 8-OHdG is one of the most used oxidative damage markers [25]. When 8-OHdG antibody staining was performed on non-irradiated sections, the levels of 8-OHdG were significantly higher in luminal cells than basal cells (Fig. 6). No significant differences were found between the TEBs and the subtending ducts (Fig. 6C).

## DISCUSSION

We classified rat mammary epithelial cells into basal, luminal progenitor and luminal mature cells based on their CK expression as well as their location (i.e. TEB or its subtending duct) and evaluated differences in the early response (1–24 h) to radiation exposure in terms of cell number in each tissue and the formation of 53BP1 foci.

Antibodies specific for CKs are widely used to identify basal cells (CK5^+^/CK14^+^) and luminal cells (CK8^+^/CK18^+^, etc.) of the mammary epithelium in mice and rats [26, 27]. The CK8 + 18 antibody we used has been reported to identify heterogeneous populations of strongly or weakly positive cells in the normal mouse mammary duct [28], yet with unknown biological significance. In the mammary gland at steady state, cells that undergo turnover in both the basal and luminal cell lineages are replaced by their respective progenitor cells, as evidenced by several cell-lineage tracing experiments [16, 17, 29]. Therein, when cells were genetically marked via expression of constitutive fluorescent protein markers and the activity of Cre recombinase directed by the CK14 promoter, only the basal cells expressed the fluorescent protein gene [17], indicating that the CK14 promoter was activated in the basal cell progenitors. Likewise, progenitor cells marked with the CK8 promoter activity were capable of producing only luminal cells [17]. In contrast, the CK18 promoter activity marks mature cells that produce very few luminal cells [17]. Thus, existing evidence suggests that CK14^+^ cells are basal cells, CK8^+^ cells are luminal cells (including progenitor and mature cells) and CK18^+^ cells are luminal mature cells. Our results confirm the presence of CK8 + 18^low^ and CK8 + 18^high^ luminal populations in the rat mammary epithelium, coinciding with CK18^−^ and CK18^+^ luminal populations, respectively. Furthermore, CK8 + 18^low^ cells were identified as containing a Ki-67^+^ population, whereas CK8 + 18^high^ cells lacked Ki-67^+^ cells. Thus, sufficient evidence supports the interpretation that CK8 + 18^low^ cells are CK18^−^, proliferation-competent luminal cell progenitors and that CK8 + 18^high^ cells are quiescent, luminal mature cells. This classification of the mammary luminal epithelial cells is simple and biologically meaningful, providing a consistent and efficient basis of studying mammary gland biology. This method is novel in that it identifies three types of cells of the rat mammary gland in situ in pathological sections, in contrast to the currently accepted method [21, 30] in which the three cell types are classified based on results of flow cytometry, which cannot discriminate between cells of the TEB and subtending duct.

Our results provide a means of classifying the mammary duct into the TEB and subtending duct as ~0.5 mm portions from the ductal terminus. In the mammary gland of young mice, Ki-67^+^ cells are more abundant in the TEB than subtending duct [23], and the thymidine analog 5-ethynyl-2′-deoxyuridine, a DNA synthesis marker, is incorporated mostly in the TEB (~0.3 mm from the ductal terminus), suggesting that this region contains proliferative cells that drive ductal elongation [31]. In our observation of mammary glands of young estrous rats, luminal mature cells were less abundant in the TEB (3.9 ± 0.9%) compared with the subtending duct (8.5 ± 4.7%), which can be interpreted as resulting from the differentiation of luminal progenitor cells during mammary gland elongation. The distribution of luminal progenitor and mature cells identified by the CK8 + 18 antibody herein is thus consistent with previous reports of experiments with mice [23, 31].

Regarding the abundance of mammary epithelial cells at the leading edge of mammary structures (i.e. TEB and subtending duct) in irradiated rats, luminal progenitor cells were the most abundant, followed by basal cells. The number of luminal progenitor cells increased transiently in the subtending duct 12 h after irradiation. It is possible that congestion occurred in the subtending duct, as shown by Paine *et al*. [18] using a mathematical model of cell dynamics in the mouse mammary gland, i.e. cells of the TEB move continuously to the subtending duct. The mechanism by which the number of luminal progenitor cells increases in response to irradiation warrants further investigation.

The proteins 53BP1 and γH2AX form foci at DSB sites immediately after radiation exposure [32] and thus are commonly used as DSB markers in vivo and in vitro [21]. We found that most radiation-induced foci disappeared within 24 h, consistent with results from previous cell culture studies [20, 33]. Our study is unique in that it followed the dynamics of a DSB marker separately in basal, luminal progenitor and luminal mature cells in both the TEB and subtending duct of the mammary gland. A previous flow cytometry study suggested that basal cells exhibit lower induction of γH2AX compared with the two luminal cell populations [21], although the method could not provide information pertaining to relative locations of cells in the mammary gland (such as the TEB and the subtending duct) with respect to progenitor cell composition. Our present observation revealed that the initial, post-irradiation abundance of DSBs was higher in luminal progenitor and mature cells than basal cells, consistent with previous results [21], and that the tendency was most obvious in the TEB by mathematical analysis. In this study, we only counted the foci on paraffin sections and did not analyze the DSB taking into account differences in 3D structure. Theoretically, the average number of foci per section (i.e. the density of foci) should be inversely proportional to the volume of the nucleus. In TEBs, there is no obvious difference in the size of basal and luminal cells (Fig. 3). In fact, it has been reported that there was no difference in the width and length between basal and luminal cells of TEBs [18]. Consequently, the results of the present study, although they do not take into account the 3D structure of the cell nucleus, are deemed to be valid. In accordance, a previous study showed that the frequency of γH2AX is higher in luminal cells than in basal cells after radiation exposure by flow cytometry analysis of primary cells [21]. Furthermore, we analyzed the time course of cell dynamics more thoroughly (i.e. post-irradiation time points 1, 3, 6, 12 and 24 h) than was done in previous studies that examined the post-irradiation time points 0.5 to 4 h [21], 2 and 24 h [34] or 1 h [35]. The results of Kudo *et al*. [21] are somewhat consistent with our present results, as mentioned above. Huper *et al*. [34] reported that γH2AX foci disappeared more quickly in basal cells than luminal cells of cultured human tissue fragments, and Coates *et al*. [35] reported that luminal cells had more γH2AX than basal cells in human tissue fragments implanted in mice; these results are somewhat inconsistent with our results. Nevertheless, these previous studies yielded qualitative results, and our more protracted time course allowed reliable quantitative analysis and revealed that cell-type dependence is governed more by the initial post-irradiation abundance of DSBs rather than repair rates.

The present study clarifies that the initial amount of radiation-induced DSBs was small in basal cells of the TEB. Ionizing radiation can damage DNA directly, i.e. via secondary electrons, or indirectly, i.e. via conversion of water molecules to reactive oxygen species (ROS) [36]. Although the former is more physical in nature, the latter could be modified biologically, with cells dealing differently with ROS. Indeed, basal cells of epithelial tissues express an abundance of the transcription factor ΔNp63α, an isoform of the Tp53-related protein Tp63 that is necessary for maintaining basal cell characteristics [37, 38]. ΔNp63α helps maintain homeostasis of the intracellular redox state by promoting the expression of enzymes that mediate the production of glutathione and cellular abundance of glutathione peroxidase 2 [39, 40], implying that basal cells are more protected than are luminal cells against radiation-induced ROS, and therefore basal cells are less likely to develop DSBs. Furthermore, in this study, we confirmed the localization of 8-OHdG, a marker of oxidative stress, in mammary tissue. The results implied that oxidative stress resistance was higher in basal cells than in luminal cells (Fig. 6). Basal cells isolated from the human mammary gland have been reported to have low levels of ROS [20]. The differential response to 8-OHdG between basal cell and luminal cell may also be related to DSB repair in response to radiation exposure. Thus, the production of antioxidants and expression of antioxidant-related enzymes and the resulting cellular response may vary from cell to cell, depending on each cell type’s tolerance to DNA damage, which may be relevant to cancer development. This is consistent with the fact that luminal cells can be the cell of origin of carcinogenesis [41, 42]. In this study, we found that DNA damage did not differ apparently between the cell types and locations in the non-irradiated tissue (Fig. 4). However, this may be due to the too small number of DSBs (<0.1 foci/nuclear section) in the non-irradiated samples to assess the relationship with the 8-OHdG level.

Biological significance of the possible different antioxidant activity between the mammary cell types remains to be elucidated. Energy biogenesis includes glycolysis and oxidative phosphorylation. We note that the glycolytic system is more functional in basal cells, whereas oxidative phosphorylation is more prominent in luminal cells [22, 43]. Glycolysis generates more ROS than the oxidative phosphorylation [44]; therefore, basal cells may have to be antioxidative to avoid excessive accumulation of cell-damaging ROS generated by glycolysis. In contrast, luminal cells use the oxidative phosphorylation more preferentially, which may keep the intracellular ROS level relatively low without antioxidants [45]. Therefore, it is plausible that differences in metabolic pathways may contribute to the susceptibility of mammary epithelial cells to DSBs. The novelty of the present study is that it separates TEBs and subtending ducts to reveal the low 8-OHdG level in basal cells compared to luminal cells in rat mammary gland. Previous studies have also focused on the differences in antioxidant capacity between basal and luminal cells; however, since these studies used flow cytometry, they did not distinguish between TEBs and ducts [20, 22]. The majority of the cells analyzed therein were likely of ductal origin. Given that TEBs are considered a source of breast cancer [41, 42], the antioxidant capacity of their cells is of greater importance than that of the ducts.

We used 1 Gy of γ-rays, a low linear energy transfer (LET) radiation, to show differential DSB generation in basal and luminal cells. Whether the present finding holds at high LET and at lower doses remains an open question. Direct actions are more important than indirect actions at high LET. Given that basal cells are more antioxidative and thereby efficiently protected from radicals produced by the indirect action, basal cells might be less protected at high LET. On the other hand, significantly higher levels of ROS and 8-OHdG have been shown in heavy-ion-irradiated than γ-irradiated mice, indicating a role of non-targeted ‘bystander’ effects or targeted secondary δ-ray effects at high LET [46, 47]. If so, basal cells might be well protected also at high LET. Regarding the dose, a recent study has reported a dose-dependent increase in intracellular ROS with a threshold near 1 Gy [48], suggesting less protection by the antioxidant capacity at low doses, whereas another study has indicated a transient increase in intracellular ROS even at 0.1 Gy [49]. Thus, whether the antioxidant capacity of basal cells in rat mammary tissue found in this study changes their response depending on dose and type of radiation should be carefully investigated in the future.

In conclusion, our results suggest that luminal progenitor and mature cells are more susceptible than are basal cells to radiation-induced DSBs in the TEB, in line with the reported hyperantioxidative state of basal cells. Moreover, we found that the repair rate did not differ among mammary epithelial cell types or their locations. The initial post-irradiation DNA damage in luminal progenitors can be considered an early event of radiation-induced carcinogenesis, and thus further study of its consequences will help explain the mechanism of mammary carcinogenesis.

## Notes

Presentation at conferences: 63rd and 64th Annual Meetings of the Japanese Radiation Research Society, and the 59th Annual Meeting on Radioisotopes and Radiation Research.
